# Activation of the STAT5 Signaling Pathway by Yiqi Jiedu Formula Induces Regulatory T Cell-Mediated Alleviation of Corneal Immunopathological Damage in Mice With Recurrent Herpes Simplex Keratitis

**DOI:** 10.3389/fphar.2021.790787

**Published:** 2022-01-21

**Authors:** Shuyu Xiao, Yang Yang, Wanhong Miao, Chunming Lyu, Jinhua Tao, Ying Yu

**Affiliations:** ^1^ Department of Ophthalmology, Shuguang Hospital Affiliated to Shanghai University of Traditional Chinese Medicine, Shanghai, China; ^2^ Experiment Center for Science and Technology, Shanghai University of Traditional Chinese Medicine, Shanghai, China; ^3^ Shanghai Eye Disease Control Center, Shanghai, China

**Keywords:** herpes simplex virus keratitis (HSK), STAT5 pathway, CD4 + CD25 + Foxp3 + Treg, YiQi JieDu(YQJD) formula, corneal immune damage

## Abstract

This study aimed to investigate the effect of Yiqi Jiedu (YQJD) formula on the repair of corneal lesions in mice with recurrent herpes simplex virus keratitis (HSK). Sixty female BALB/c mice were randomly divided into three groups: a normal control group (Naive), a recurrence model group (Re), and a YQJD group. After inducing recurrence by ultraviolet irradiation, the ocular surfaces of different groups of mice were observed using a slit lamp and photographed, and ocular surface scores were calculated. The abundance of CD4^+^CD25^+^Foxp3^+^ regulatory T (Treg) cells was determined by flow cytometry in peripheral blood and spleen cells. The CD4^+^Foxp3^+^ Tregs were assessed by immunofluorescence in the cornea. The levels of the cytokines IL-10 and TGF-β in serum and splenocyte culture supernatants were detected by enzyme-linked immunosorbent assay. Furthermore, the activation status of the STAT5 signaling pathway was examined by protein blotting, and the effect of YQJD on Treg cells through inhibition of the STAT5 pathway was observed *in vitro*. YQJD alleviated corneal inflammation by enhancing the STAT5 signaling pathway, thereby promoting the differentiation of CD4^+^CD25^+^Foxp3^+^ Treg cells, increasing the levels of anti-inflammatory cytokines such as IL-10 and TGF-β, and maintaining immune tolerance. YQJD increased the proportion of CD4^+^Foxp3^+^ Treg cells; also, in the cornea, YQJD inhibited the aggregation of macrophages and CD4^+^ cells and reduced the proportion of Th17 cells and other pro-inflammatory cells. Moreover, YQJD promoted the secretion of IL-4 to protect the cornea, leading to the mitigation of corneal immunopathological damage. YQJD reduced corneal lesions in recurrent HSK mice by stimulating Treg cells, inducing immune tolerance, and inhibiting corneal immunopathological responses via modulation of the STAT5 signaling pathway.

## Introduction

Herpes simplex virus type 1 (HSV-1) is a prevalent viral pathogen that infects most of the world’s population (Rowe et al., 2013). When infecting the cornea by interacting with host cell surface receptors through its glycoproteins, the HSV-1 virus induces a chronic immune-inflammatory response known as herpes simplex virus keratitis (HSK) (Koujah et al., 2019). The direct pathogenicity of the virus and the potent immune response triggered by viral proteins induce inward vascular growth, leukocyte infiltration, and corneal stromal and endothelial damage, leading to corneal clouding, edema, and neovascularization (Fukuda et al., 2008). Moreover, recurrent HSK can lead to progressive and irreversible corneal scar formation, which can ultimately cause blindness (Rowe et al., 2013). HSV-1 infections can be divided into primary and recurrent. After initial infection, HSV-1 spreads to the trigeminal ganglion (TG), where it becomes latent. Subsequently, after being activated by stimuli such as fever, menstruation, exertion, trauma, ultraviolet (UV) radiation, and some immunodeficiency diseases (Al-Dujaili et al., 2011), the virus migrates from the TG to the corneal area, inducing recurrent corneal infections that can affect vision.

Recurrent HSK is associated not only with the probability of viral reactivation, but also with the expression of viral and host proteins, the accumulation and the kinetics of specific innate and adaptive immune effectors, and the activation of inflammatory responses (Perng et al., 2016). In particular, immune responses triggered by the activation of latent HSV-1 are the main cause of recurrent HSK corneal lesions (Wang et al., 2020). After reactivation, HSV-1 viruses begin to replicate in the infected corneal epithelium and bind to Toll-like receptors (TLRs) on the cell surface, inducing a series of immune responses and signaling pathways that stimulate the production of inflammatory cells; these, in turn, trigger progressive infiltration of pro-inflammatory factors and chemokines into the stroma, thereby aggravating corneal lesion, and even causing blindness. Therefore, the corneal inflammatory response caused by HSV-1 ocular infection and chronic lesions induced by effector T cells can lead to blindness (Bhela et al., 2017). Unfortunately, the efficacy of anti-HSV therapy is limited (Sumbria, et al., 2021). In fact, no specific treatment for recurrent HSK has yet been developed, and long-term antiviral therapy based on acyclovir only reduces recurrence rates by approximately 40% (Ziyaeyan et al., 2007). Moreover, hepatic and renal toxicity associated with long-term consumption of oral antiviral drugs should not be ignored. Thus, prevention and repair of corneal damage after recurrence may be a promising new approach for the treatment of recurrent HSK.

Nevertheless, repairing corneal lesions and scars is a challenging task, since immune regulation and homeostasis are critical to determine the corneal response to treatment (Foulsham et al., 2018). In addition, harmful T-cell effectors that lead to corneal scarring (CS) deserve in-depth investigation (Jaggi et al., 2018). A previous study has revealed that HSV-1 infection and anti-HSV-1 antibodies modulate the activity of regulatory T (Treg) cells (Ciurkiewicz et al., 2020). However, little research has been conducted on the role of Treg cells in HSV-1 entry into latency or reactivation from dormancy. Mott et al. (2014) reported that the negative effect of CD8α^+^ dendritic cells on the replication of viral RNA molecules and latency-associated transcripts (LAT) under T-cell depletion led to a large accumulation of latent viral genome in the TG of intraocularly infected mice, and to an increased recurrence rate. Notably, bilateral disease, recurrent infections, and CS are observed more frequently in immunocompromised patients (Liesegang, 2001). Moreover, a recent study showed that treatment of virus-induced inflammatory responses with anti-IL-27 antibodies increased the number of CD4^+^CD25^+^Foxp3^+^ Treg cells and improved corneal tissue damage during relapses (Xia et al., 2019). Overall, previous research has demonstrated that Tregs play a protective role by maintaining homeostasis *in vivo*, enhancing immune tolerance, improving corneal damage, and preventing the onset of autoimmune diseases. Therefore, Tregs are considered protective immunomodulatory mediators that control the release of inflammatory factors and chemokines against viral invasion into the cornea (Veiga-Parga et al., 2012). Thus, it is important to study the role of CD4^+^CD25^+^Foxp3^+^ regulatory T cells in the modulation of corneal lesions during HSK relapse.

Treg cells, expressing the transcription factor Foxp3 as a characteristic molecular marker, play an essential role in maintaining immune homeostasis and preventing the disruption of peripheral self-tolerance (Georgiev et al., 2019). CD4^+^CD25^+^Foxp3^+^ T cells, a subpopulation of suppressor T cells, primarily mediate peripheral immune tolerance (Deng et al., 2019). The identity of Tregs and the production of their major regulatory factors are determined by Foxp3 expression; in particular, the expression of CD25, a direct target of Foxp3, is critical for Treg proliferation, survival, and Foxp3 expression (Cheng et al., 2011). In addition, the activation of Foxp3 depends on the STAT5 signaling pathway (Whitehouse et al., 2017; Apert et al., 2018; Fan et al., 2018), a key regulatory pathway for the differentiation of parental CD4^+^ T cells into Tregs (Shi et al., 2018). Therefore, in this study we aimed to test whether the Yiqi Jiedu (YQJD) formula plays a role in activating Tregs through the STAT5 signaling pathway to help reduce corneal lesions via overall immune regulation.

The YQJD formula is composed of Huang Qi (*Astragalus mongholicus Bunge.*), Bai Zhu (*Atractylodes macrocephala Koidz.*), Fang Feng [*Saposhnikovia divaricata* (Turcz. ex Ledeb.) Schischk.], Jin Yin Hua (*Lonicera japonica Thunb.*), Pu Gong Ying (*Taraxacum mongolicum Hand.-Mazz.*), Da Qing Ye (*Isatis tinctoria subsp. Tinctoria.*), Zi Cao [*Arnebia euchroma (Royle ex Benth.) I.M.Johnst.*], Chan Tui (*Cryptotympana pustulata Fabricius.*), Fu Ling {Wolfiporia extensa (Peck) Ginns [syn. Poria cocos (Schw.)]}, Sheng Di Huang [*Rehmannia glutinosa (Gaertn.) DC.*] and Chai Hu (*Bupleurum chinense DC.*). YQJD is a prescription drug based on clinical experience, consisting of Yupingfeng (YPF) powder, which promotes a healthy body Qi, supplemented by *Lonicera japonica Thunb.* and *Taraxacum mongolicum Hand.-Mazz.* for heat clearing and detoxification, and Yin-nourishing drugs such as Wolfiporia extensa (Peck) Ginns (syn. Poria cocos (Schw.)), *Rehmannia glutinosa (Gaertn.) DC.*, and *Bupleurum chinense DC.* to harmonize the Yin-Yang balance. YPF, an ancient Chinese herbal decoction, was developed from Dan-Xi Xin Fa by Zhu Dan-Xi of the Chinese Yuan Dynasty, and has been used in clinical practice for the treatment of cold, flu, and inflammation-related diseases for several centuries, due to its immunomodulatory properties (Fan et al., 2017). Moreover, heat-clearing and detoxifying drugs such as *Arnebia euchroma (Royle ex Benth.) I.M.Johnst.*, *Isatis tinctoria subsp. Tinctoria.* supplemented with *Lonicera japonica Thunb.* and *Taraxacum mongolicum Hand.-Mazz.* (from the Dictionary of Tumor Prescriptions) can exert anti-inflammatory, antibacterial, and antiviral effects (Muluye et al., 2014; Su et al., 2021), as well as regulate immune inflammatory responses. Finally, *Cryptotympana pustulata Fabricius.*, Wolfiporia extensa (Peck) Ginns [syn. Poria cocos (Schw.)], *Rehmannia glutinosa (Gaertn.) DC.* (Zhou et al., 2019), and *Bupleurum chinense DC.* have immunomodulatory (Li et al., 2019), anti-inflammatory, and antiviral properties (Yang et al., 2017). Early clinical studies in our department and clinical observations by other researchers have shown that the YQJD formula, prescribed to strengthen body resistance and eliminate pathogenic factors, was safe and effective for the treatment and prevention of recurrent HSK. Indeed, the YQJD formula could relieve the symptoms of patients, improve vital signs, and repair the cornea to a certain extent. However, no studies have yet been conducted to reveal its mechanism of action. Nevertheless, in-depth investigation of the mechanism by which the YQJD formula alleviates corneal lesions in mice with recurrent HSK is important for its application in clinical treatment.

Recurrent HSK ophthalmopathy can be fatal to the cornea, leading to visual damage. However, our previous clinical study revealed that the administration of YQJD formula before the onset of recurrence could improve the ocular surface symptoms of patients. In this study, we assessed whether the YQJD formula could activate the STAT5 signaling pathway in mice, induce CD4^+^CD25^+^Foxp3^+^ Treg cells to exert immune tolerance, inhibit the effects of systemic immune inflammation on corneal damage, and stimulate Tregs to repair corneal damage. Our results provide a scientific basis for the use of the YQJD formula to prevent and treat recurrent HSK.

## Materials and Methods

### Drugs Preparation

All granules for the *in vivo* experiments were purchased from Jiangyin Tianjiang Pharmaceutical Co. Ltd. The clinical drug dose and medicinal part of each botanical drug are listed in Table 1. YQJD formula at a dry weight ratio of 7:4:4:4:5:5:3:3:3:4:3 was provided by the Shanghai Shuguang Hospital. The equivalent dose was calculated according to the clinical human dose (60 g per day) and the surface area ratio of the human to the animal. The YQJD formula (10 doses) was added with 1.5 L distilled water and heated for 10 min under continuous stirring at 100°C. Then, the concentration of the YQJD formula was diluted to 1100 mg/ml. For *in vivo* experiments, 0.1 ml/10 g of YQJD was administered intragastrically, whereas 0.11 g/ml was used to treat cells *in vitro*.

**TABLE 1:** The composition of the YQJD formula.

Botanical drug	Latin scientific name	Officinal part	Family	Dosage (g)
Astragali radix (*Huang Qi*)	*Astragalus mongholicus Bunge*	Root	Lamiaceae	20
Atractylodis macrocephalae rhizoma (*Bai Zhu*)	*Atractylodes macrocephala Koidz*	Rhizome	Asteraceae	12
Saposhnikoviae radix (*Fang Feng*)	*Saposhnikovia divaricata* (Turcz. ex Ledeb.) Schischk	Root	Umbelliferae cruciferae	12
Flos lonicerae (*Jin Yin Hua*)	*Lonicera japonica Thunb*	Bud	Caprifoliaceae	12
Taraxaci herba (*Pu Gong Ying*)	*Taraxacum mongolicum Hand.-Mazz*	Whole grass	Asteraceae	15
Folium isatidis (*Da Qing Ye*)	*Isatis tinctoria subsp. Tinctoria*	Leaf	Cruciferae	15
Radix arnebiae (*Zi Cao*)	*Arnebia euchroma (Royle ex Benth.) I.M.Johnst*	Rhizome	Boraginaceae	10
Periostracum cicada (*Chan Tui*)	*Cryptotympana pustulata Fabricius*	Shell	Cicadidae	9
Poria (*Fu Ling*)	Wolfiporia extensa (Peck) Ginns [syn. Poria cocos (Schw.)]	Sclerotium Root	Polyporaceae	10
Rehmannia glutinosa (*Sheng Di Huang*)	*Rehmannia glutinosa (Gaertn.) DC.*	Root	Scrophulariaceae	12
Bupleurum (*Chai Hu*)	*Bupleurum chinense DC.*	Root	Umbelliferae	9

### Analysis of the Chemical Compositions of the YQJD Formula by High-Resolution Mass Spectrometry

Samples of YQJD formula were analyzed using an ultra-high performance liquid chromatography with quadrupole time-of-flight mass spectrometry (UPLC-Q TOF/MS) system (Waters H Class, Waters Technology Co., Ltd., and Sciex TriPle TOF 4600 LC/MS, Shanghai Aibo Caisi Analyt. Instruments Trading Co., Ltd.). Both positive and negative ion modes were applied for parallel reaction monitoring, and mobile phases consisting of 0.1% formic acid in water (A) and acetonitrile (B) were used to separate analytes on a Waters ACQUITY UPLC ® HSS T3 column (2.1 × 100 mm, 1.8 µm). Gradient elution was set as follows: 0–5 min, 0% B; 5–15 min, 0%–15% B; 15–30 min, 15%–60% B; 30–35 min, 60%–95% B; 35–40 min, 95%–95% B; and 40 min, 95%–95% B. The flow rate was 0.3 ml/min, the injection volume was 3 μL, and the column temperature was 30°C. Thirty-four compounds were identified by comparing the multilevel mass spectra of the samples with available information from the Natural Products High Resolution Mass Spectrometry database (Natural Products HR-MS/MS SPectral Library 1.0.1 database) and related literature (Lee et al., 2017) (Supplementary Material).

### Mice and Ethics Statement

Sixty specific pathogen-free, five-week-old BALB/c female mice were purchased from Shanghai Siple-Bikai Laboratory Animal Co., Ltd. [No. 20180006001983, License No. SCXK (Shanghai) 2018-0006]. All mice were housed at the Animal Experiment Center of the Shanghai University of Traditional Chinese Medicine. This study was approved by the Ethics Committee of the Shanghai University of Traditional Chinese Medicine (approval number, PZSHUTCM180222039) and was conducted in accordance with the Declaration on Animal Use of the American Association for Research in Vision and Ophthalmology. All mice were housed at room temperature (22–24°C) under a 12-h light-dark cycle, at a relative humidity of 40–60%, and with free access to standard food and water.

### Animal Model and Treatments

Sixty female mice were randomly divided into a control group (Naive), a recurrence model (Re) control group, and a YQJD group, with 20 mice in each group. An HSV-1 suspension [5 μL, 1 × 10^6^ plaque-forming units (PFU)] was dripped into the right eye of each mouse belonging to the Re and YQJD groups according to the method described in the literature (BenMohamed et al., 2016); next, full absorption of the viral suspension was induced by gently massaging the eyes with sterile cotton swabs. After 3 weeks, the mice of the YQJD group were administered 0.1 ml/10 g of YQJD twice a day for 2 weeks. After 5 weeks, the corneas of mice from each group were examined under a slit lamp microscope, and spontaneous recurrence of HSK was excluded. The mice of the Re group received UV treatment. Specifically, the right eye of each mouse belonging to the Re group was irradiated with a UV lamp (UV-B-302 nm) for 2.75 min, at an irradiation dose of 2.104 mJ/cm^2^. Changes in the ocular surface and staining of mice were observed daily before and 1–3 days after UV lamp irradiation. Three days after UV lamp irradiation, aseptic spleen collection, blood collection, and eyeball enucleation were performed in Re mice, for subsequent parameter analysis.

### Performance and Assessment of Ocular Surface Lesions

1) Scoring standard for corneal stromal opacities:

0: Normal, not diffuse; no haze; 1.0–1.9: Significant edema; slight haze; iris clearly visible; 2.0–2.9: Gross edema; stromal swelling; cloudy, diffuse; anterior chamber visible; iris visible; 3.0–3.9: Severe stromal edema; very cloudy, anterior chamber invisible; pupillary border no longer distinct; 4: Opaque corneas; anterior chamber structure not visible.

2) Score for blepharitis:

0: Normal; 1.0–1.9: Slight eyelid swelling; 2.0–2.9: Medium blepharoedema; eyeball secretions; 3.0–3.9: Severe swelling of the eyelids; moderate exfoliation and lesions of the skin around the eyes; 4: Severe swelling of the eyelids; severe exfoliation and lesions of the skin around the eyes.

Total ocular surface lesion score = corneal stroma opacity score + blepharitis score.

### Western Blotting

Spleen tissue was lysed by ultrasound; next, spleen protein samples were extracted and stored in a refrigerator at −80°C. Western blotting was performed to detect the protein expression of STAT5/p-STAT5 in the spleen, as follows: after preparing the gel kit, samples (15 μL) were loaded and the electrophoresis solution was added. Electrophoresis was then carried out at 120 V for 1 h. After pouring fresh transfer solution, polyvinylidene fluoride (PVDF) membranes were transferred at a constant current of 250 mA for 1 h and then blocked with bovine serum albumin (BSA) for 2 h. The PVDF membranes were then washed with Tris-buffered saline containing 0.1% Tween-20 (TBST) three times and incubated overnight at 4°C with different primary antibodies (anti-p-STAT5, 1:2000, CST, 4322; anti-STAT5, 1:2000, CST, 94205; anti-Neuropilin-1, 1:1500, CST, 3725; and anti-β-actin, 1:2000, CST, 4970). Next, the PVDF membranes were washed with TBST three times and incubated with horseradish peroxidase (HRP)-conjugated secondary antibodies (CST) for approximately 2 h at room temperature. Finally, the blots were developed with a chemiluminescence reagent using an enhanced chemiluminescence kit (BeyoECL Plus. P0018M, Beyotime). Data were analyzed using ImageJ software.

### Quantitative PCR

Corneal total RNA (1 μg) was reverse transcribed and quantified at 260 nm; next, the transcriptional levels of *Il-17a*, *Cd11c*, *Tgf-β*, and *Il-4* were evaluated by quantitative PCR. The following primers were designed using NCBI Prime-blast and synthesized by Suzhou Jinweizhi: 1) *Il-17a*, NM_010,552.3 (amplicon length, 195 bp); 2) *Cd11c*, NM_001363984.1 (amplicon length, 143 bp); 3) *Tgf-β*, NM_011577.2 (amplicon length, 99 bp); and 4) *Il-4*, NM_021283.2 (amplicon length, 117 bp). *Gapdh* (NM_001289726.1; amplicon length, 123 bp) was used to standardize gene expression levels. The copy number of each reaction product was determined by normalizing the threshold cycle of each sample against a standard curve. To calculate the fold change in expression, ΔCt values against the internal control were computed, and the 2^−ΔΔCt^ method was then applied to calculate the fold change of gene expression compared to that in the Naive control group.

### Immunofluorescence Assay

Mouse eyeballs were immersed in 4% formaldehyde (self-provisioned in the laboratory), dehydrated in alcohol (at increasing concentrations of 30, 50, and 70% for 60 min each time; and of 85, 95, 100, and 100% for 30 min each time), and then embedded in xylene and paraffin. Paraffin-embedded tissue blocks were cut into serial 5-μm sections. For antigen retrieval, the slides were immersed in ethylenediaminetetraacetic acid (EDTA) antigen retrieval buffer (pH 8.0), maintained at sub-boiling temperature for 8 min, let stand for 8 min, and then subjected again to sub-boiling temperature for 7 min. Care was taken to avoid evaporation of the buffer solution. The slides were then air-cooled and washed three times with phosphate buffered saline (PBS; pH 7.4) in a Rocker device for 5 min each time. After blocking with PBS containing 3% BSA at room temperature for 30 min, sections were incubated at 4°C overnight with primary antibodies against F480 (1:1,000, Servicebio, GB11027), CD4 (1:1,000, Abcam, ab183685), CD8 (1:1,000, Abcam, ab217344), and Foxp3 (1:100, Santa Cruz Biotechnology, sc53896). Then, the liquid was removed carefully. Next, the sections were incubated with a secondary antibody (1:300, Servicebio, GB21303) at room temperature for 50 min in the dark. Subsequently, the sections were incubated with DAPI solution at room temperature for 10 min and kept in the dark. A spontaneous fluorescence quenching reagent was added and the slides were incubated for 5 min, and then washed under running tap water for 10 min. The liquid was removed carefully, and then a cover slip with an anti-fade mounting medium was placed on the slides. Blue DAPI fluorescence was elicited by UV excitation at 330–380 nm and detected at an emission wavelength of 420 nm; green FITC fluorescence was elicited at an excitation wavelength of 465–495 nm and detected at an emission wavelength of 515–555 nm; and red CY3 fluorescence was elicited at an excitation wavelength of 510–560 nm and detected at an emission wavelength of 590 nm.

### Flow Cytometry Analysis

Peripheral blood (150 μL) was collected and added to 3 ml of hemolysin. The samples were centrifuged after a 10-min incubation and washed once with PBS. Surface staining was performed by adding 2 μL of anti-CD3 (PE-Cy7), anti-CD4 (BV786), or anti-CD25 (AF488) antibody (eBioscience) to each tube, and by subsequently washing with PBS. Then, 1 ml of Foxp3 Fix/Perm liquid (1:3 configuration, eBioscience™ Foxp3/Transcription Factor Staining Buffer Set, 00-5523-00) was added to fix the samples, which were incubated at 4°C for 45 min. After centrifugation at 500 × g for 5 min, the supernatant was removed. After the addition of 1× Perm liquid (2 ml), each sample was washed and the supernatant was removed. Intracellular staining was performed by incubating the samples with anti-Foxp3 antibody (1 μL, eBioscience, PE) for 30 min, and by subsequently washing each sample with 1× Perm liquid (2 ml) and adding the fixative solvent. Finally, the samples were analyzed using Flow Jo10.

### Enzyme-Linked Immunosorbent Assay

Specific kits against IL-10 (Boster Bio, EK0417) and TGF-β (Boster Bio, EK0515) were used to detect cytokine levels before and after HSK recurrence in serum and spleen cells of mice. The procedure was performed according to the manufacturer’s instructions.

### Quantification of Serum Drug Levels

Ten healthy Sprague Dawley rats (200 ± 15 g) were selected and divided into two groups, each consisting of five rats: a YQJD group and a blank group. Blood was collected from the abdominal aorta under aseptic conditions 1 h later and then centrifuged at 3000 rpm for 10 min at 4°C. The separated serum was inactivated in a water bath at 56°C for 30 min and then refrigerated at −80°C.

### Splenocyte Culture

CD4^+^CD25^+^ Treg cells were isolated using blank mouse spleen cells with a CD4^+^CD25^+^ Treg cell isolation kit (mouse, MACS, 130-091-041). The cells were then cultured in RPMI 1640 complete medium at 37°C in a humidified atmosphere containing 5% CO_2_. This experiment included three groups: a YQJD group (reaching a drug serum concentration of 10%), a YQJD + STAT5 inhibitor (STAT5-IN-1, Selleckchem, SH-4-54) treatment group, and a mock-treated blank control group. Treg cells (2  × 10^5^ cells/mL) were plated on RPMI 1640 supplemented with 35 μL of anti-CD3/CD28 antibodies and 5 ng/ml IL-2 for 72 h. The YQJD + STAT5 inhibitor group was treated with 50 μM STAT5 inhibitor. Finally, cultured cells were collected for western blotting and flow cytometry.

### Statistical Analysis

Data are expressed as the mean ± SD. Statistical analysis was performed using GraphPad Prism 7 statistical software (GraphPad Software, Inc., La Jolla, CA, United States, serial number: GPS-0320559-LFUL-95242) and SPSS 22.0 software (IBM Corp., Armonk, NY, United States). Unpaired Student’s *t*-test was used to compare two groups, while one-way analysis of variance (ANOVA) with post-hoc Bonferroni test or Kruskal-Wallis test was performed to compare more than two groups. Statistical significance was set at *p* < 0.05.

## Results

### Yiqi Jiedu Inhibits the Expression of Corneal Pro-inflammatory Factors and Promotes the Expression of Anti-inflammatory Factors to Alleviate Corneal Damage Caused by Recurrent HSK

**FIGURE 1:**
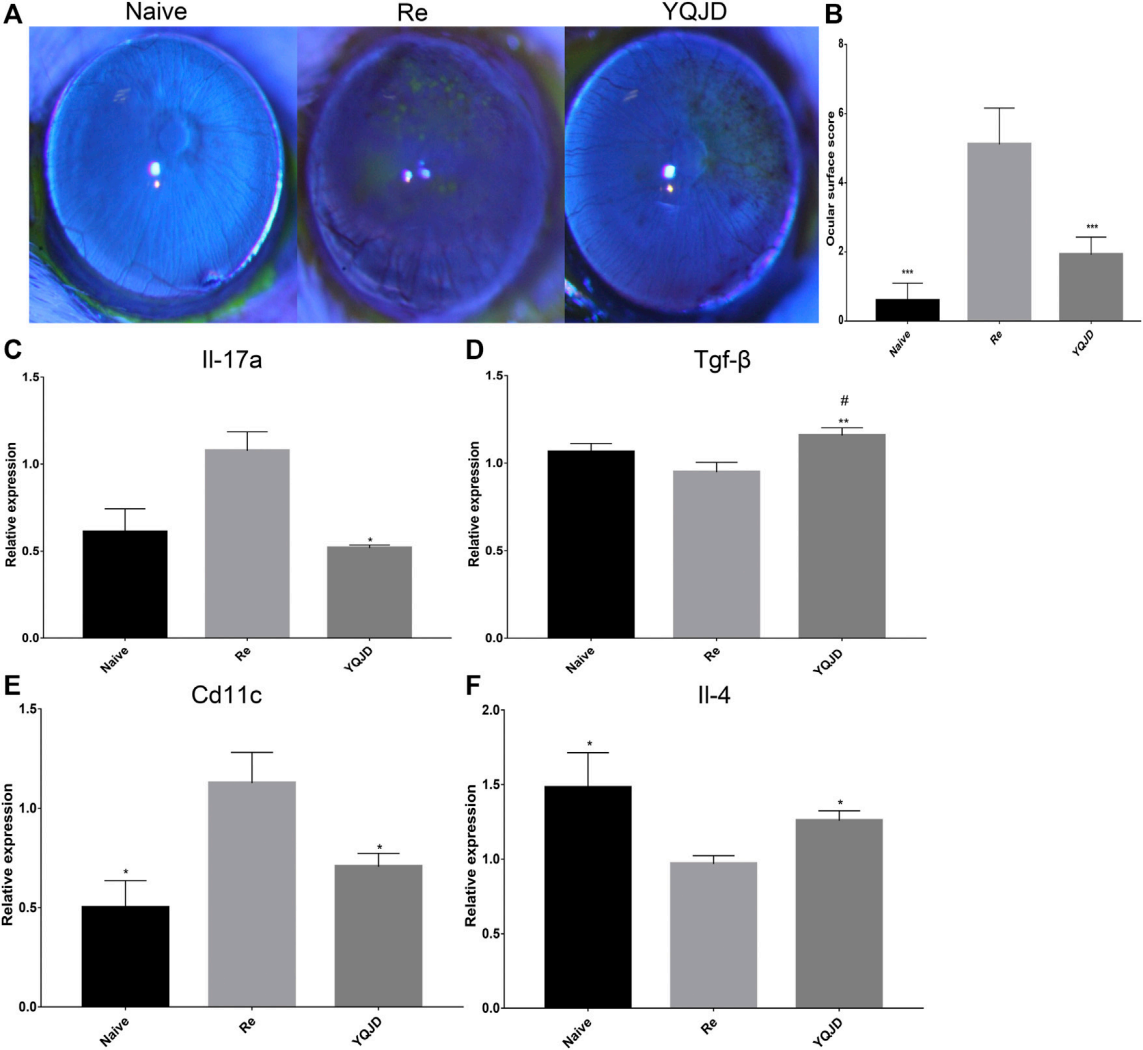
Effects of YQJD treatment on the ocular surface score and cytokine gene expression. **(A)** Representative images of stained mice ocular surfaces 3 days after UV irradiation. Sodium fluorescein staining was carried out as follows: the operator grasped the mouse’s ears and neck skin with the thumb and index finger of the left hand. The ring finger and little finger of the left hand were used to clamp the skin on the back and the tail, while the right hand was kept steady and with minimal shaking. The cornea was stained with 10 g/L sodium fluorescein and examined under a slit lamp microscope. **(B)** Ocular surface scores of mice 3 days after UV irradiation. **(C–F)** Expression of **(C)** *Il-17*, **(D)** *Tgf-β*, **(E)** *Cd11c*, and **(F)** *Il-4* in the mouse cornea. Data are expressed as the mean ± SD. *p < 0.05, **p < 0.01, and ***p < 0.001 vs. the Re group; #*p* < 0.05 vs. the Naive group; *n* = 6 for ocular surface scoring; *n* = 3 for RT-qPCR experiments.

Sodium fluorescein staining revealed clear corneal staining and the absence of surface loss and neovascularization in the Naive group. Moreover, compared to the Re group, clearer corneal staining, less pronounced neovascularization, and much less significant surface loss were observed in the YQJD group. Consistently, ocular surface scores were lower in the Naive and YQJD formula groups than in the Re group (Figures 1A, B). Consistently, ocular surface scores were lower in the Naive and YQJD formula groups than in the Re group.

Furthermore, the effect of the YQJD formula on the expression levels of cytokines in the corneas of recurrent HSK mice was evaluated. The expression levels of various cytokine-encoding genes, namely *Cd11c*, *Il-17a*, *Il-4*, and *Tgf-β*, were measured by reverse transcription quantitative PCR (RT-qPCR) in mixed corneal samples. Compared to those in the Re group, *Il-17a* levels were significantly decreased in the YQJD group (Figure 1C), whereas *Tgf-β* and *Il-4* levels were significantly increased in the YQJD group (Figures 1D, F), and *Cd11c* levels were significantly decreased in the YQJD and Naive groups (Figure 1E). However, the expression levels of these four cytokine genes did not differ significantly between the YQJD group and the Naive group. Early treatment with YQJD formula inhibited the expression of several HSK pro-inflammatory cytokines and increased that of cytokines that exert anti-inflammatory functions.

### Yiqi Jiedu Promotes Corneal Treg Accumulation and Suppresses F480 and CD4 Infiltration

**FIGURE 2:**
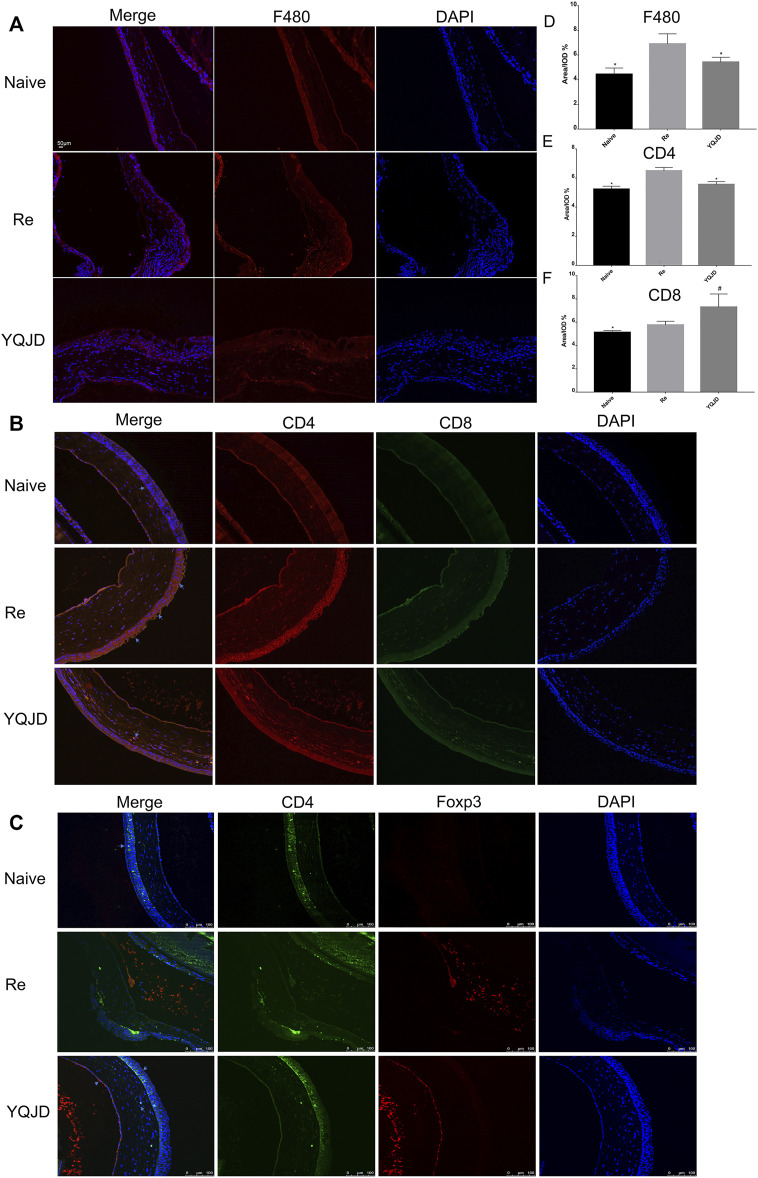
Effects of YQJD treatment on the expression of F480, CD4, and CD8 in the cornea. **(A)** Expression of F480. **(B)** Expression of CD4 (red) and CD8 (green). **(C)** Expression of CD4 (green) and Foxp3 (red). **(D)** Statistical analysis of F480 expression. **(E)** Statistical analysis of CD4 expression. **(F)** Statistical analysis of CD8 expression. The data are expressed as the mean ± SD of three independent experiments. *p < 0.05 vs. the Re group; #*p* < 0.05 vs. the Naive group.

The expression of F480, CD4, and CD8 in the cornea of different groups of mice was investigated by immunofluorescence to explore the effect of the YQJD formula on the corneal immune response of mice (Figures 2A–C). Compared to those in the YQJD treatment group and the Naive group, the expression levels of F480 and CD4 in the cornea were significantly higher in the Re group. Moreover, the levels of CD4^+^Foxp3^+^ Treg cells in the YQJD group were higher than those in the Re group, suggesting that YQJD can alleviate pathological inflammatory responses in the cornea of recurrent HSK mice (Figures 2D–G). The field size was 10 × 20 μm. Image-Pro Plus software was used to perform a semi-quantitative analysis of the fluorescence signal.

### The Yiqi Jiedu Formula Induces CD4^+^CD25^+^Foxp3^+^ Treg Cells and the Expression of Anti-inflammatory Factors in Peripheral Blood and Spleen of Mice with Recurrent HSK

**FIGURE 3:**
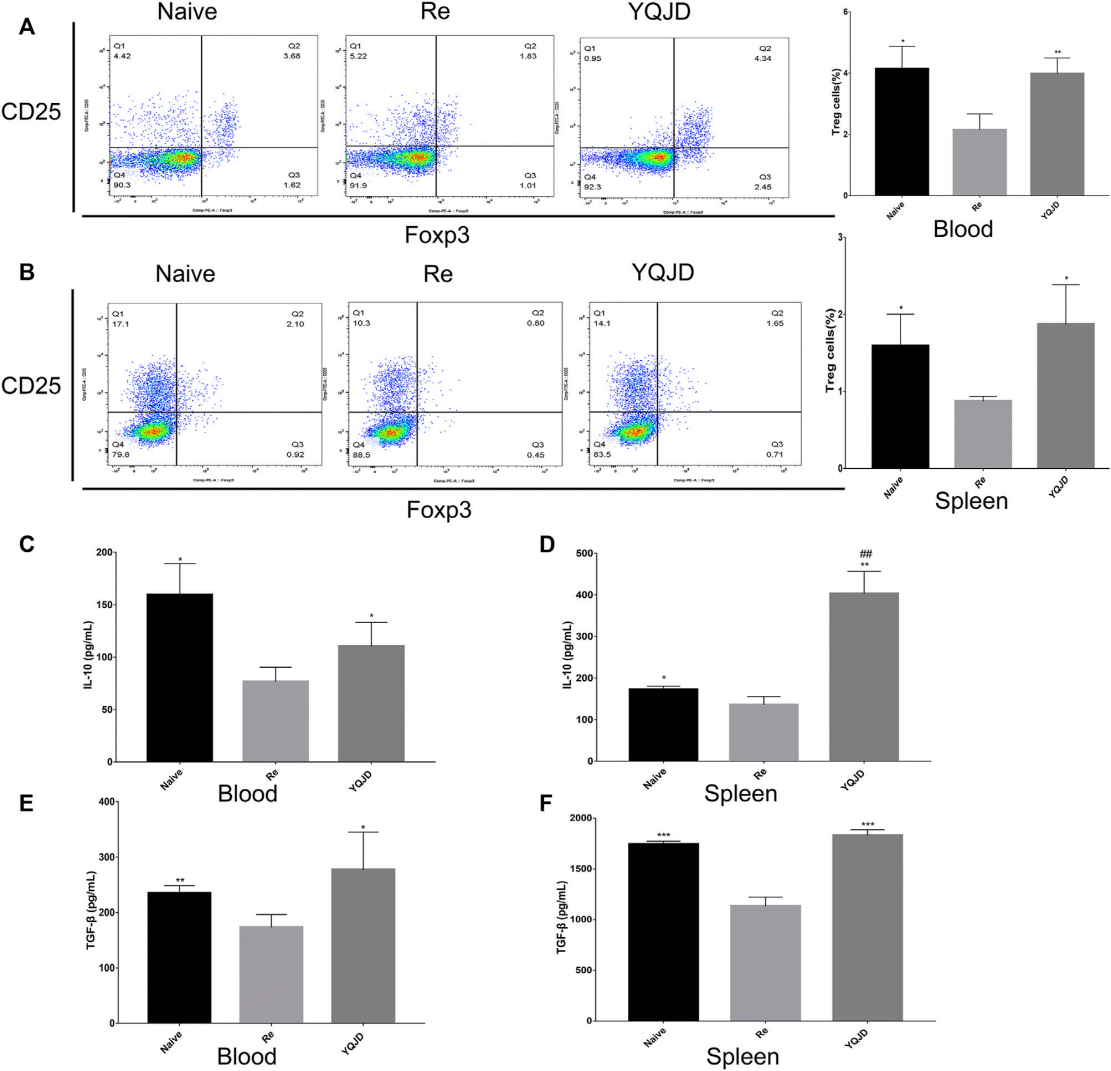
Effects of YQJD treatment on the immune response in peripheral blood and spleen of recurrent HSK mice. **(A)** Levels of CD4^+^CD25^+^Foxp3^+^ Tregs in peripheral blood. **(B)** Levels of CD4^+^CD25^+^Foxp3^+^ Tregs in the spleen. **(C)** Expression of IL-10 in peripheral blood. **(D)** Expression of IL-10 in spleen cells. **(E)** Expression of TGF-β in peripheral blood. **(F)** Expression of TGF-β in spleen cells. The data are expressed as the mean ± SD of five independent experiments. *p < 0.05, **p < 0.01, and ***p < 0.001 vs. the Re group; *#p* < 0.05 and *##p* < 0.01 *vs*. the Naive group.

+Peripheral blood and spleen samples were collected from mice of the different groups to examine the effects of the YQJD formula on the immune response and cytokine levels of mice after recurrent infection. The levels of CD4^+^CD25^+^Foxp3^+^ Treg cells in peripheral blood and spleen cells of the YQJD and Naive groups were significantly higher than those of the Re group (Figures 3A,B). Moreover, the expression of IL-10 and TGF-β in the peripheral blood of mice in the YQJD group was higher than that of mice in the Re group (*p* < 0.05, *p* < 0.05), but did not differ significantly from that of mice in the Naive group; in addition, the expression of IL-10 and TGF-β in the peripheral blood of mice in the Naive group was higher than that of mice in the Re group (*p* < 0.05, *p* < 0.01; Figures 3C,E). Similarly, the expression of IL-10 and TGF-β in the spleen cells of mice in the YQJD group was higher than that of mice in the Re group (*p* < 0.01, *p* < 0.001). Also, the expression of IL-10 and TGF-β in splenocytes of mice in the Naive group was higher than that of mice in the Re group (*p* < 0.05, *p* < 0.001; Figures 3D,F).

### The Yiqi Jiedu Formula Protects From Corneal Damage After Recurrent HSK by Enhancing p-STAT5 and Neuropilin-1 Levels

**FIGURE 4:**
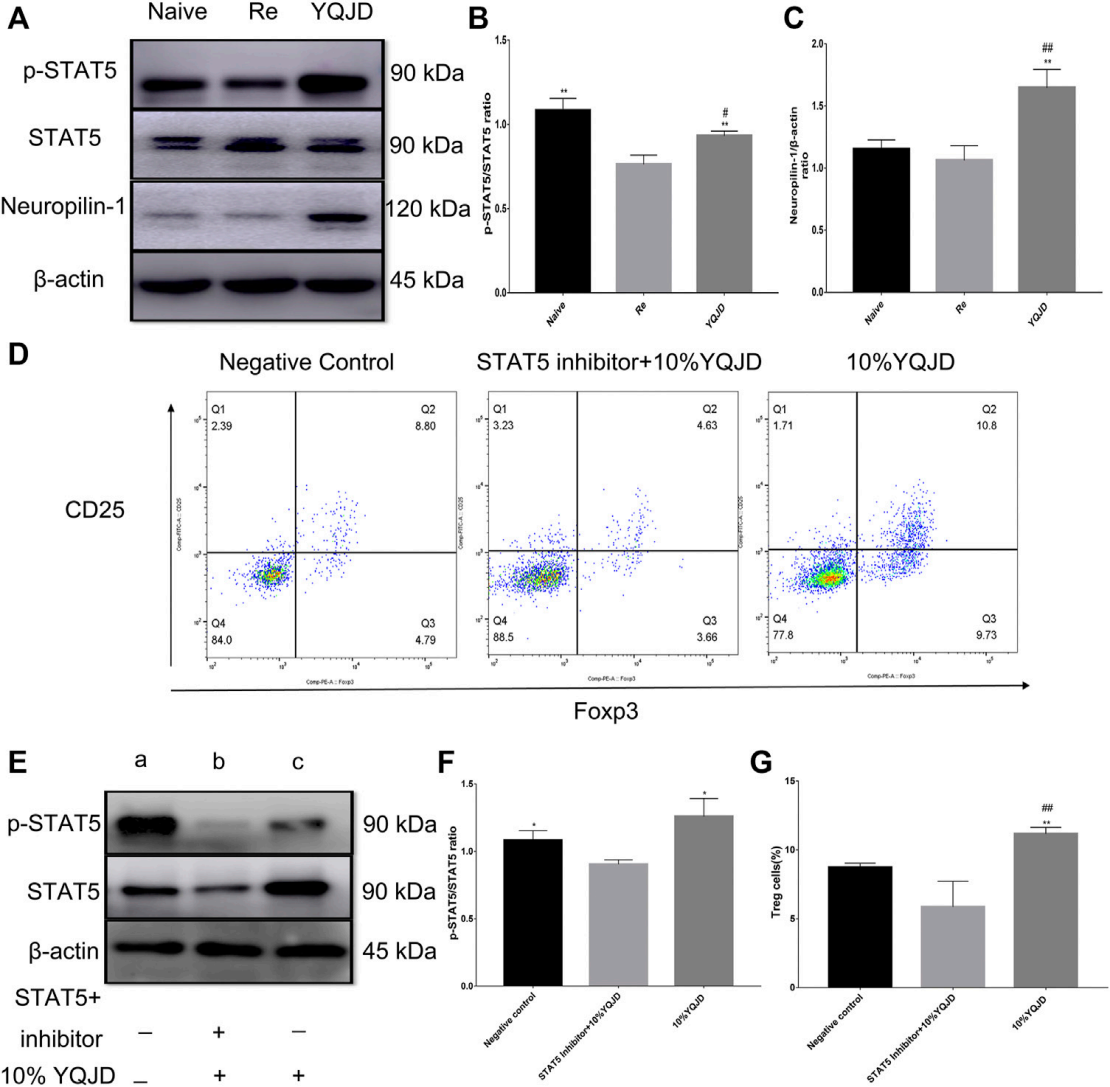
Effects of YQJD treatment on the STAT5 signaling pathway in spleen cells of recurrent HSK mice. **(A)** Relative expression of p-STAT5/STAT5 and Neuropilin-1 in spleen tissues. **(B,C)** Statistical analysis of p-STAT5/STAT5 and Neuropilin-1 expression. **(D)** Expression of CD4^+^CD25^+^Foxp3^+^ Treg cells in an *in vitro* culture of mouse spleen cells. **(E)** Relative expression of p-STAT5/STAT5 in an *in vitro* culture of mouse spleen cells. **(F)** Statistical analysis of p-STAT5/STAT5 expression in an *in vitro* culture of mouse spleen tissue (a, NC group; b, STAT5 inhibitor +10% YQJD group; c, 10% YQJD group). **(G)** Statistical analysis of CD4^+^CD25 + Foxp3^+^ Treg cell levels in an *in vitro* culture of mouse spleen cells. The data are expressed as the mean ± SD of three independent experiments. *p < 0.05 and **p < 0.01 vs. the Re group; ##*p* < 0.01 vs. the Naive group; *p < 0.05 and **p < 0.01 vs. the STAT5 inhibitor + 10% YQJD group; #*p* < 0.05 and ##*p* < 0.01 vs. the NC group.

Compared to those of the Re group, p-STAT5 levels in the YQJD and Naive groups were significantly increased. In addition, the expression of p-STAT5 in the YQJD group was significantly higher than that in the Naive group (Figures 4A,B). Moreover, the levels of Neuropilin-1 increased significantly in the YQJD group, compared to those of both the Naive group and the Re group (Figure 4C). In summary, the expression of STAT5 and Neuropilin-1 was upregulated by the YQJD formula. CD4^+^CD25^+^Foxp3^+^ Treg cells from the spleens of BALB/c mice were cultured and sorted *in vitro* to verify the mechanism by which the YQJD formula increases Treg levels. Compared to those in the NC group and the STAT5 inhibitor group, the levels of Tregs in the 10% YQJD group were significantly enhanced (Figures 4D,G). Moreover, the levels of p-STAT5 in the 10% YQJD and NC groups were significantly higher than those in the STAT5 inhibitor group (Figures 4E,F). In conclusion, STAT5 protein levels and Treg levels were both upregulated by the YQJD formula.

## Discussion

The ocular surface is a unique mucosal immune zone with anatomical, physiological, and immunological features that act in synergy to establish a particularly tolerant microenvironment. It has been reported that ocular infection by HSV-1 can lead to chronic T cell-mediated inflammatory lesions in the cornea (Bhela et al., 2017). The severity of these lesions is influenced by the balance of different CD4^+^ T-cell subsets, since these are the main coordinators of immunopathological damage (Lobo et al., 2019). Notably, the severity and duration of lesions depend to a greater extent on the balance of T-cell functional subsets rather than on the presence of non-lymphocytes such as neutrophils and macrophages or impairments in neoangiogenesis and neurological function (Jaggi et al., 2020). In particular, it has been observed that lesions are reduced in presence of cells with predominant regulatory functions (Rajasagi and Rouse, 2019). In contrast, lesions are more severe when pro-inflammatory Th1 and Th17 cells are predominant owing to an impaired Treg cell balance (Rouse and Suvas, 2004). In summary, CD4^+^ T cells, CD8^+^ T cells, and Tregs, the major coordinators of immunopathological lesions, play essential roles in the pathogenesis of HSK and corneal injury.

In this study, the corneas of mice were assessed daily for signs of lesions after relapse induction. Compared to those of mice in the Re group, the corneas of mice in the YQJD group were more transparent and presented less neovascularization and lower ocular surface scores, indicating that the YQJD formula mitigated macrophage infiltration in the cornea after HSV-1 infection. IL-17, a key pro-inflammatory cytokine, promotes the immune-inflammatory response to HSK by stimulating the production of pro-inflammatory factors and neutrophil-derived chemokines through the regulation of the secretion of corneal stromal fibroblasts (Maertzdorf et al., 2002). Consistently, anti-IL-17 antibodies can effectively suppress such delayed-type hypersensitivity response and significantly reduce lesion severity in HSK mouse models (Xia et al., 2013). Conversely, IL-4 secreted by Tregs downregulates the expression of inflammatory factors and prevents the formation of HSV-1-induced corneal scars, and, more importantly, reduces viral replication in the cornea (Osorio et al., 2002). Altogether, these findings suggest that YQJD may modulate the expression of anti-inflammatory factors to mitigate the corneal immunopathological response in recurrent HSK mice.

The therapeutic effects of pharmacotherapy on the ocular surface have been well documented. In fact, a series of studies have verified the protective role of Tregs against HSV-1-induced immune-mediated inflammatory processes (Veiga-Parga et al., 2012). In particular, Foxp3^+^ Tregs are central regulators of immune homeostasis and tolerance (Do et al., 2016). Compared to downregulating the number of CD4^+^ T cells to attenuate Th1 cell responses (Xia et al., 2010), stabilizing Tregs *via* Neuropilin-1 treatment has been shown to be more effective in curbing the development of HSK lesions (Sidaway, 2017). In addition, CD4^+^CD25^+^Foxp3^+^ Tregs protect the cornea against severe damage, whereas their depletion accelerates HSK progression (Suvas et al., 2004). Moreover, CD4^+^CD25^+^Foxp3^+^ Tregs can secrete IL-10, and IL-10 treatment has been shown to reduce neutrophil infiltration into the cornea and improve the degree of corneal damage (Keadle and Stuart, 2005). Consequently, some researchers have proposed to treat HSK by upregulating IL-10 expression (Rajasagi et al., 2012), which can significantly reduce the incidence of HSK and control the degree of inflammation (Tumpey et al., 1998; Sarangi et al., 2008). These results suggest that the YQJD formula promotes the differentiation of CD4^+^CD25^+^Foxp3^+^ Tregs and the expression of anti-inflammatory cytokines in the cornea of mice with recurrent HSK, thereby reducing the severity of the lesions.

The differentiation of Tregs mainly depends on the activation of STAT5 signaling (Cheng et al., 2013). In fact, STAT5 can directly bind to the conserved regions of the *Foxp3* gene to promote Foxp3 expression. In this study, we observed that YQJD could effectively promote the activation of the STAT5 signaling pathway, which may subsequently induce the differentiation of CD4^+^CD25^+^Foxp3^+^ Tregs, indicating a suppressive immunopathological effect of the YQJD formula. Similarly, a single administration of 2,3,7,8-tetrachlorodibenzo-p-dioxin by injection has been reported to significantly reduce the severity of HSK lesions in mice (Veiga-Parga et al., 2011). Corneal scar formation and angiogenesis are the result of a chronic inflammatory response against HSV antigens, and intraperitoneal administration of galectin-1 significantly reduced the severity of HSK lesions as well as neointimal formation (Rajasagi et al., 2012). Moreover, systemic administration of Tregs has been applied as a treatment for uveitis (Gregoire et al., 2016), suggesting that this exerts significant effects on the cornea.

HSK, a chronic immunopathological lesion, has been shown to severely impair visual quality (Rajasagi et al., 2017). In particular, over the course of blinding inflammation of the ocular surface, an inadequate immune response increases disease susceptibility, whereas an overreaction induces corneal clouding through inflammatory cell damage; this indicates that balanced immunopathological responses are crucial for preventing corneal clouding, scar formation, and permanent vision loss (Xia et al., 2019). Tregs and the cytokines IL-10 and TGF-β play a protective role by maintaining homeostasis, enhancing immune tolerance, and preventing the onset of autoimmune diseases. Tregs can also reduce susceptibility to disease throughout the body, control the release of inflammatory cytokines and chemokines, and prevent viral replication (Veiga-Parga et al., 2012). Conversely, anti-inflammatory cytokines such as IL-4 can adjust the ratio of anti-inflammatory cells and their activity to reduce the severity of corneal lesions (Varanasi et al., 2017). In summary, our results demonstrated that YQJD can enhance Treg differentiation and reduce corneal lesions; these effects are potentially mediated by the STAT5 signaling pathway. Early administration of YQJD formula can activate the STAT5 signaling pathway to increase the proportion of Tregs while inhibiting the accumulation pro-inflammatory factors and inducing that of anti-inflammatory factors, thereby leading to improved immune tolerance and reduced severity of corneal lesions.

## Conclusion

The mechanism which the YQJD formula alleviates corneal inflammatory damage caused by recurrent HSK has been elucidated in this study. The prevention and treatment of recurrent herpes simplex keratitis provide new immune ideas and strategies. We will explore the influence of YQJD on aqueous humor, limbal region blood vessels and the corneal immune microenvironment. It is helpful to clarify the mechanism of drug action. At the same time, further research is made on the main active ingredients in the YQJD prescription.

## Data Availability

The raw data supporting the conclusion of this article will be made available by the authors, without undue reservation.

## References

[B1] Al-Dujaili L. J. Clerkin P. P. Clement C. McFerrin H. E. Bhattacharjee P. S. Varnell E. D. (2011). Ocular Herpes Simplex Virus: How Are Latency, Reactivation, Recurrent Disease and Therapy Interrelated? Future Microbiol. 6, 877–907. 10.2217/FMB.11.73 21861620 PMC3403814

[B2] Apert C. Romagnoli P. van Meerwijk J. P. M. (2018). IL-2 and IL-15 Dependent Thymic Development of Foxp3-Expressing Regulatory T Lymphocytes. Protein Cell 9, 322–332. 10.1007/s13238-017-0425-3 28540653 PMC5876181

[B3] BenMohamed L. Osorio N. Khan A. A. Srivastava R. Huang L. Krochmal J. J. (2016). Prior Corneal Scarification and Injection of Immune Serum Are Not Required before Ocular HSV-1 Infection for UV-B-Induced Virus Reactivation and Recurrent Herpetic Corneal Disease in Latently Infected Mice. Curr. Eye Res. 41, 747–756. 10.3109/02713683.2015.1061024 26398722 PMC5349863

[B4] Bhela S. Varanasi S. K. Jaggi U. Sloan S. S. Rajasagi N. K. Rouse B. T. (2017). The Plasticity and Stability of Regulatory T Cells during Viral-Induced Inflammatory Lesions. J. Immunol. 199, 1342–1352. 10.4049/jimmunol.1700520 28710254 PMC5548616

[B5] Cheng G. Yu A. Dee M. J. Malek T. R. (2013). IL-2R Signaling Is Essential for Functional Maturation of Regulatory T Cells during Thymic Development. J. Immunol. 190, 1567–1575. 10.4049/jimmunol.1201218 23315074 PMC3563871

[B6] Cheng G. Yu A. Malek T. R. (2011). T-cell Tolerance and the Multi-Functional Role of IL-2R Signaling in T-Regulatory Cells. Immunol. Rev. 241, 63–76. 10.1111/j.1600-065X.2011.01004.x 21488890 PMC3101713

[B7] Ciurkiewicz M. Herder V. Beineke A. (2020). Beneficial and Detrimental Effects of Regulatory T Cells in Neurotropic Virus Infections. Int. J. Mol. Sci. 21. 10.3390/ijms21051705 PMC7084400 32131483

[B8] Deng G. Song X. Fujimoto S. Piccirillo C. A. Nagai Y. Greene M. I. (2019). Foxp3 Post-translational Modifications and Treg Suppressive Activity. Front. Immunol. 10, 2486. 10.3389/fimmu.2019.02486 31681337 PMC6813729

[B9] Do J. S. Visperas A. Sanogo Y. O. Bechtel J. J. Dvorina N. Kim S. (2016). An IL-27/Lag3 axis Enhances Foxp3+ Regulatory T Cell-Suppressive Function and Therapeutic Efficacy. Mucosal Immunol. 9, 137–145. 10.1038/mi.2015.45 26013006 PMC4662649

[B10] Fan M. Y. Low J. S. Tanimine N. Finn K. K. Priyadharshini B. Germana S. K. (2018). Differential Roles of IL-2 Signaling in Developing versus Mature Tregs. Cell Rep 25, 1204–e4. 10.1016/j.celrep.2018.10.002 30380412 PMC6289175

[B11] Fan W. Zheng P. Wang Y. Hao P. Liu J. Zhao X. (2017). Analysis of Immunostimulatory Activity of Polysaccharide Extracted from Yu-Ping-Feng *In Vitro* and *In Vivo* . Biomed. Pharmacother. 93, 146–155. 10.1016/j.biopha.2017.05.138 28628831

[B12] Foulsham W. Coco G. Amouzegar A. Chauhan S. K. Dana R. (2018). When Clarity Is Crucial: Regulating Ocular Surface Immunity. Trends Immunol. 39, 288–301. 10.1016/j.it.2017.11.007 29248310 PMC5880704

[B13] Fukuda M. Deai T. Higaki S. Hayashi K. Shimomura Y. (2008). Presence of a Large Amount of Herpes Simplex Virus Genome in Tear Fluid of Herpetic Stromal Keratitis and Persistent Epithelial Defect Patients. Semin. Ophthalmol. 23, 217–220. 10.1080/08820530802111366 18584558

[B14] Georgiev P. Charbonnier L.-M. Chatila T. A. (2019). Regulatory T Cells: the Many Faces of Foxp3. J. Clin. Immunol. 39, 623–640. 10.1007/s10875-019-00684-7 31478130 PMC6754763

[B15] Grégoire S. Terrada C. Martin G. H. Fourcade G. Baeyens A. Marodon G. (2016). Treatment of Uveitis by *In Situ* Administration of Ex Vivo-Activated Polyclonal Regulatory T Cells. J. Immunol. 196, 2109–2118. 10.4049/jimmunol.1501723 26826251

[B17] Jaggi U. Wang S. Tormanen K. Matundan H. Ljubimov A. V. Ghiasi H. (2018). Role of Herpes Simplex Virus Type 1 (HSV-1) Glycoprotein K (gK) Pathogenic CD8+ T Cells in Exacerbation of Eye Disease. Front. Immunol. 9, 2895. 10.3389/fimmu.2018.02895 30581441 PMC6292954

[B18] Jaggi U. Yang M. Matundan H. H. Hirose S. Shah P. K. Sharifi B. G. (2020). Increased Phagocytosis in the Presence of Enhanced M2-like Macrophage Responses Correlates with Increased Primary and Latent HSV-1 Infection. Plos Pathog. 16, e1008971. 10.1371/journal.ppat.1008971 33031415 PMC7575112

[B19] Keadle T. L. Stuart P. M. (2005). Interleukin-10 (IL-10) Ameliorates Corneal Disease in a Mouse Model of Recurrent Herpetic Keratitis. Microb. Pathog. 38, 13–21. 10.1016/j.micpath.2004.09.003 15652291

[B20] Koujah L. Suryawanshi R. K. Shukla D. (2019). Pathological Processes Activated by Herpes Simplex Virus-1 (HSV-1) Infection in the Cornea. Cell Mol Life Sci 76, 405–419. 10.1007/s00018-018-2938-1 30327839 PMC6349487

[B21] Lee S. M. Jeong J. S. Kwon H. J. Hong S. P. (2017). Quantification of Isoflavonoids and Triterpene Saponins in Astragali Radix, the Root of Astragalus Membranaceus, via Reverse-phase High-Performance Liquid Chromatography Coupled with Integrated Pulsed Amperometric Detection. J. Chromatogr. B Analyt Technol. Biomed. Life Sci. 1070, 76–81. 10.1016/j.jchromb.2017.10.046 29102246

[B22] Li X. He Y. Zeng P. Liu Y. Zhang M. Hao C. (2019). Molecular Basis for Poria Cocos Mushroom Polysaccharide Used as an Antitumour Drug in China. J. Cell Mol Med 23, 4–20. 10.1111/jcmm.13564 30444050 PMC6307810

[B23] Liesegang T. J. (2001). Herpes Simplex Virus Epidemiology and Ocular Importance. Cornea 20, 1–13. 10.1097/00003226-200101000-00001 11188989

[B24] Lobo A. M. Agelidis A. M. Shukla D. (2019). Pathogenesis of Herpes Simplex Keratitis: The Host Cell Response and Ocular Surface Sequelae to Infection and Inflammation. Ocul. Surf. 17, 40–49. 10.1016/j.jtos.2018.10.002 30317007 PMC6340725

[B25] Maertzdorf J. Osterhaus A. D. Verjans G. M. (2002). IL-17 Expression in Human Herpetic Stromal Keratitis: Modulatory Effects on Chemokine Production by Corneal Fibroblasts. J. Immunol. 169, 5897–5903. 10.4049/jimmunol.169.10.5897 12421973

[B26] Mott K. R. Allen S. J. Zandian M. Ghiasi H. (2014). Coregulatory Interactions Among CD8α Dendritic Cells, the Latency-Associated Transcript, and Programmed Death 1 Contribute to Higher Levels of Herpes Simplex Virus 1 Latency. J. Virol. 88, 6599–6610. 10.1128/JVI.00590-14 24672046 PMC4054370

[B27] Muluye R. A. Bian Y. Alemu P. N. (2014). Anti-inflammatory and Antimicrobial Effects of Heat-Clearing Chinese Herbs: A Current Review. J. Tradit Complement. Med. 4, 93–98. 10.4103/2225-4110.126635 24860732 PMC4003708

[B28] Osorio Y. Sharifi B. G. Perng G. Ghiasi N. S. Ghiasi H. (2002). The Role of T(H)1 and T(H)2 Cytokines in HSV-1-Induced Corneal Scarring. Ocul. Immunol. Inflamm. 10, 105–116. 10.1076/ocii.10.2.105.13982 12778346

[B29] Perng G. C. Osorio N. Jiang X. Geertsema R. Hsiang C. Brown D. (2016). Large Amounts of Reactivated Virus in Tears Precedes Recurrent Herpes Stromal Keratitis in Stressed Rabbits Latently Infected with Herpes Simplex Virus. Curr. Eye Res. 41, 284–291. 10.3109/02713683.2015.1020172 25859798 PMC5349859

[B30] Rajasagi N. K. Bhela S. Varanasi S. K. Rouse B. T. (2017). Frontline Science: Aspirin-Triggered Resolvin D1 Controls Herpes Simplex Virus-Induced Corneal Immunopathology. J. Leukoc. Biol. 102, 1159–1171. 10.1189/jlb.3HI1216-511RR 28584076 PMC5636045

[B31] Rajasagi N. K. Rouse B. T. (2019). The Role of T Cells in Herpes Stromal Keratitis. Front. Immunol. 10, 512. 10.3389/fimmu.2019.00512 30941142 PMC6433787

[B32] Rajasagi N. K. Suryawanshi A. Sehrawat S. Reddy P. B. Mulik S. Hirashima M. (2012). Galectin-1 Reduces the Severity of Herpes Simplex Virus-Induced Ocular Immunopathological Lesions. J. Immunol. 188, 4631–4643. 10.4049/jimmunol.1103063 22467659 PMC3338323

[B33] Rouse B. T. Suvas S. (2004). Regulatory Cells and Infectious Agents: Détentes Cordiale and Contraire. J. Immunol. 173, 2211–2215. 10.4049/jimmunol.173.4.2211 15294929

[B34] Rowe A. M. St. Leger A. J. Jeon S. Dhaliwal D. K. Knickelbein J. E. Hendricks R. L. (2013). Herpes Keratitis. Prog. Retin. Eye Res. 32, 88–101. 10.1016/j.preteyeres.2012.08.002 22944008 PMC3529813

[B35] Sarangi P. P. Sehrawat S. Suvas S. Rouse B. T. (2008). IL-10 and Natural Regulatory T Cells: Two Independent Anti-inflammatory Mechanisms in Herpes Simplex Virus-Induced Ocular Immunopathology. J. Immunol. 180, 6297–6306. 10.4049/jimmunol.180.9.6297 18424753

[B16] Shi H. Liu C. Tan H. Li Y. Nguyen T. M. Dhungana Y. (2018). Hippo Kinases Mst1 and Mst2 Sense and Amplify IL-2R-STAT5 Signaling in Regulatory T Cells to Establish Stable Regulatory Activity. Immunity 49, 899-e6. 10.1016/j.immuni.2018.10.010 30413360 PMC6249059

[B36] Sidaway P. (2017). Immunotherapy: Neuropilin-1 Is Required for Treg Stability. Nat. Rev. Clin. Oncol. 14, 458. 10.1038/nrclinonc.2017.90 28607516

[B37] Su X. Zhu Z. H. Zhang L. Wang Q. Xu M. M. Lu C. (2021). Anti-inflammatory Property and Functional Substances of Lonicerae Japonicae Caulis. J. Ethnopharmacol 267, 113502. 10.1016/j.jep.2020.113502 33189843

[B38] Sumbria D. Berber E. Rouse B. T. (2021). Supplementing the Diet with Sodium Propionate Suppresses the Severity of Viral Immuno-Inflammatory Lesions. J. Virol. 95 (4), e02056–20. 10.1128/JVI.02056-20 33208449 PMC7851545

[B39] Suvas S. Azkur A. K. Kim B. S. Kumaraguru U. Rouse B. T. (2004). CD4+CD25+ Regulatory T Cells Control the Severity of Viral Immunoinflammatory Lesions. J. Immunol. 172, 4123–4132. 10.4049/jimmunol.172.7.4123 15034024

[B40] Tumpey T. M. Cheng H. Yan X. T. Oakes J. E. Lausch R. N. (1998). Chemokine Synthesis in the HSV-1-Infected Cornea and its Suppression by Interleukin-10. J. Leukoc. Biol. 63, 486–492. 10.1002/jlb.63.4.486 9544579

[B41] Varanasi S. K. Reddy P. B. J. Bhela S. Jaggi U. Gimenez F. Rouse B. T. (2017). Azacytidine Treatment Inhibits the Progression of Herpes Stromal Keratitis by Enhancing Regulatory T Cell Function. J. Virol. 91 (7), e02367–16. 10.1128/JVI.02367-16 28100624 PMC5355591

[B42] Veiga-Parga T. Suryawanshi A. Mulik S. Giménez F. Sharma S. Sparwasser T. (2012). On the Role of Regulatory T Cells during Viral-Induced Inflammatory Lesions. J. Immunol. 189, 5924–5933. 10.4049/jimmunol.1202322 23129753 PMC3518750

[B43] Veiga-Parga T. Suryawanshi A. Rouse B. T. (2011). Controlling Viral Immuno-Inflammatory Lesions by Modulating Aryl Hydrocarbon Receptor Signaling. Plos Pathog. 7, e1002427. 10.1371/journal.ppat.1002427 22174686 PMC3234248

[B44] Wang L. Wang R. Xu C. Zhou H. (2020). Pathogenesis of Herpes Stromal Keratitis: Immune Inflammatory Response Mediated by Inflammatory Regulators. Front. Immunol. 11, 766. 10.3389/fimmu.2020.00766 32477330 PMC7237736

[B45] Whitehouse G. Gray E. Mastoridis S. Merritt E. Kodela E. Yang J. H. M. (2017). IL-2 Therapy Restores Regulatory T-Cell Dysfunction Induced by Calcineurin Inhibitors. Proc. Natl. Acad. Sci. U S A. 114, 7083–7088. 10.1073/pnas.1620835114 28584086 PMC5502598

[B46] Xia L. Tan T. Li Y. Zhong Q. Shi M. (2019). Blockade of IL-27 Signaling Ameliorates Herpes Stromal Keratitis with Upregulated CD4+ Foxp3+ Regulatory T Cells Influx in Mice. Indian J. Ophthalmol. 67, 1821–1828. 10.4103/ijo.IJO_1780_18 31638041 PMC6836587

[B47] Xia L. Zhang S. Cao Z. Hu Y. Yang H. Wang D. (2013). Interleukin-17 Enhanced Immunoinflammatory Lesions in a Mouse Model of Recurrent Herpetic Keratitis. Microbes Infect. 15, 126–139. 10.1016/j.micinf.2012.10.017 23159245

[B48] Xia L. Zhang S. Zhou J. Li Y. (2010). A Crucial Role for B and T Lymphocyte Attenuator in Preventing the Development of CD4+ T Cell-Mediated Herpetic Stromal Keratitis. Mol. Vis. 16, 2071–2083. 21042564 PMC2965573

[B49] Yang F. Dong X. Yin X. Wang W. You L. Ni J. (2017). Radix Bupleuri: A Review of Traditional Uses, Botany, Phytochemistry, Pharmacology, and Toxicology. Biomed. Res. Int. 2017, 1–22. 10.1155/2017/7597596 PMC5448051 28593176

[B50] Zhou Y.-X. Zhang R.-Q. Rahman K. Cao Z.-X. Zhang H. Peng C. (2019). Diverse Pharmacological Activities and Potential Medicinal Benefits of Geniposide. Evidence-Based Complement. Altern. Med. 2019, 1–15. 10.1155/2019/4925682 PMC6500620 31118959

[B51] Ziyaeyan M. Alborzi A. Japoni A. Kadivar M. Davarpanah M. A. Pourabbas B. (2007). Frequency of Acyclovir-Resistant Herpes Simplex Viruses Isolated from the General Immunocompetent Population and Patients with Acquired Immunodeficiency Syndrome. Int. J. Dermatol. 46, 1263–1266. 10.1111/j.1365-4632.2007.03449.x 18173520

