# Supplementary material for: Activation of the STAT5 Signaling Pathway by Yiqi Jiedu Formula Induces Regulatory T Cell-Mediated Alleviation of Corneal Immunopathological Damage in Mice With Recurrent Herpes Simplex Keratitis
Source: Front Pharmacol. 2022 Jan 21;12:790787. doi: 10.3389/fphar.2021.790787 (PMC8814580; doi:10.3389/fphar.2021.790787)
Supplement: Supplementary file 1 [file DataSheet1.docx]

UPLC-Q-Orbitrap HRMS analysis of YQJD
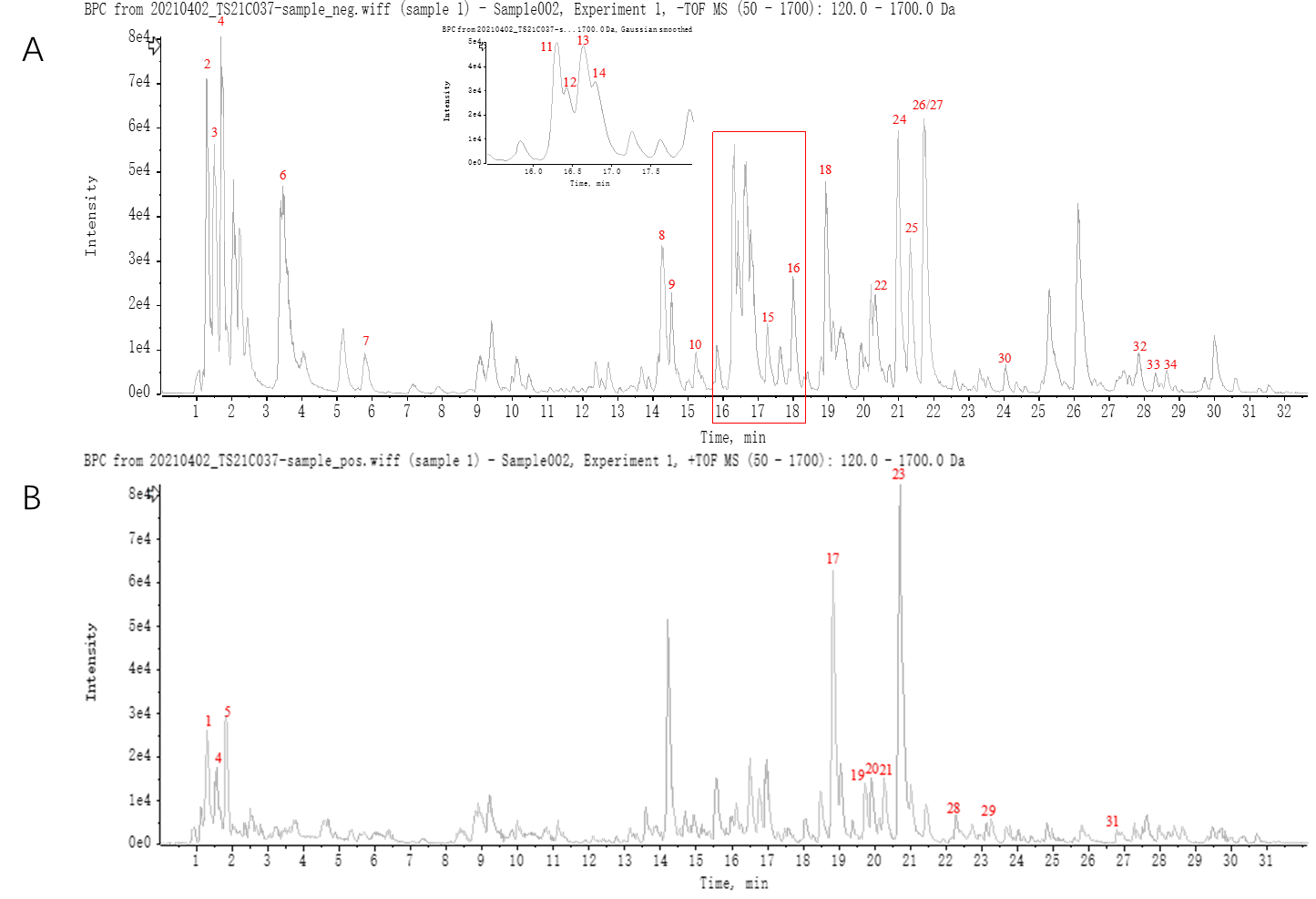


1. UHPLC-HRMS base peak ion chromatogram (BPC) of stock extract sample-negative ion mode；B. UHPLC-HRMS base peak ion chromatogram (BPC)-positive ion mode of stock extract sample

Table 1 Standard spectral information of compounds in YQJD

| No. | Time  （min） | Mode | *m*/*z*  Actual value | *m*/*z*  Theoretical value | Quality deviation  ppm | formula | English name | MS/MS data | adscription |
| --- | --- | --- | --- | --- | --- | --- | --- | --- | --- |
| 1 | 3.47 | [M-H]^-^ | 191.0197 | 191.0197 | -0.1 | C6H8O7 | Citric acid | 191.0187;111.0086;87.0084;85.0293;57.0344 | all |
| 2 | 5.81 | [M-H]^-^ | 243.0621 | 243.0623 | -0.7 | C9H12N2O6 | Uridine | 200.0578;152.0358;110.0247;82.0303;66.0357 | all |
| 3 | 14.28 | [M-H]^-^ | 353.0879 | 353.0878 | 0.3 | C16H18O9 | Neochlorogenic acid | 353.0855;191.0554;179.0374;135.0445 | Flos Lonicerae |
| 4 | 14.69 | [M-H]^-^ | 137.0245 | 137.0244 | 0.6 | C7H6O3 | Protocatechnic aldehyde | 137.0246;136.0163;108.0213;92.0265 | Flos Lonicerae, Taraxaci Herba |
| 5 | 15.23 | [M-H]^-^ | 375.1302 | 375.1297 | 1.4 | C16H24O10 | Loganic acid | 375.1281;213.0761;169.0857;151.0750;113.0244 | Flos Lonicerae |
| 6 | 16.30 | [M-H]^-^ | 389.1094 | 389.1089 | 1.2 | C16H22O11 | Secologanoside | 389.1082;345.1190;209.0462;183.0658;165.0559  121.0665;89.0249;69.0351;59.0142 | Flos Lonicerae |
| 7 | 16.44 | [M-H]^-^ | 353.0882 | 353.0878 | 1.1 | C16H18O9 | Chlorogenic acid | 191.0554 | Flos Lonicerae, Taraxaci Herba |
| 8 | 16.64 | [M-H]^-^ | 373.1145 | 373.1140 | 1.3 | C16H22O10 | Secologanic acid | 193.0506;149.0604;119.03551;97.0295;89.0245 | Flos Lonicerae |
| 9 | 16.81 | [M-H]^-^ | 353.0886 | 353.0878 | 2.2 | C16H18O9 | Cryptochlorogenic acid | 353.0878;191.0565;179.0358;173.0464;135.0459 | Flos Lonicerae |
| 10 | 17.33 | [M-H]^-^ | 179.0353 | 179.0350 | 1.8 | C9H8O4 | Caffeic acid | 135.0459;134.0377 | all |
| 11 | 18.01 | [M-H]^-^ | 403.1262 | 403.1246 | 4.0 | C16H22O9 | Sweroside | 403.1232;195.0650;179.0545;125.0241;81.0341 | Flos Lonicerae |
| 12 | 18.84 | [M+H]^+^ | 469.1714 | 469.1704 | 3.5 | C22H28O11 | Prim-O-glucosylcimifugin | 469.1704;307.1177;290.1140;261.1119;235.0599 | Saposhnikoviae Radix |
| 13 | 18.95 | [M-H]^-^ | 403.1263 | 403.1246 | 4.3 | C17H24O11 | Secoxyloganin | 371.0975;223.0615;121.0300;89.0249;59.0138 | Flos Lonicerae |
| 14 | 19.74 | [M+H]^+^ | 447.1287 | 447.1286 | 0.3 | C22H22O10 | Calycosin-7-glucoside | 285.0754;270.0512;253.0487 | Astragali radix |
| 15 | 19.92 | [M+H]^+^ | 433.1134 | 433.1129 | 1.1 | C21H20O10 | Isovitexin | 397.0931;379.0825;337.0717;313.0718;283.0610 | Folium Isatidis |
| 16 | 20.28 | [M+H]^+^ | 307.1185 | 307.1176 | 2.9 | C16H18O6 | Cimifugin | 307.1186;289.1075;259.0597;235.0601;221.0440 | Saposhnikoviae Radix |
| 17 | 20.33 | [M-H]^-^ | 473.0727 | 473.0725 | 0.3 | C22H18O12 | Chicoric acid | 311.0440;293.0318;219.0310;179.0362;149.0104;  135.0459 | Taraxaci Herba |
| 18 | 20.71 | [M+H]^+^ | 453.1743 | 453.1755 | -2.7 | C22H28O10 | 4'-O-beta-Glucopyranosyl-5-O-Methylvisamminol | 453.1755;291.1224;273.1119;231.0651 | Saposhnikoviae Radix |
| 19 | 20.99 | [M-H]^-^ | 515.1201 | 515.1195 | 1.2 | C25H24O12 | Isochlorogenic acid C | 515.1203;353.0857;191.0556;179.0352;173.0457 | Flos Lonicerae |
| 20 | 21.34 | [M-H]^-^ | 515.1207 | 515.1195 | 2.3 | C25H24O12 | Isochlorogenic acid A | 515.1204;353.0884;191.0568;179.0357;135.0458 | Flos Lonicerae |
| 21 | 21.74 | [M-H]^-^ | 515.1218 | 515.1195 | 4.5 | C25H24O12 | Isochlorogenic acid B | 515.1208;353.0878;191.0561;179.0353;173.0458 | Flos Lonicerae |
| 22 | 21.86 | [M-H]^-^ | 187.0980 | 187.0976 | 2.2 | C9H16O4 | Azelaic acid | 187.0970;125.0970;123.0815;97.0664 | Flos Lonicerae |
| 23 | 22.29 | [M+H]^+^ | 431.1332 | 431.1337 | -1.1 | C22H22O9 | Ononin | 269.0800;254.0552;237.0537 | Astragali radix |
| 24 | 23.28 | [M+H]^+^ | 439.1590 | 439.1599 | -2.0 | C21H26O10 | Sec-O-Glucosylhamaudol | 277.1079;259.0973;217.0503;205.0504 | Saposhnikoviae Radix |
| 25 | 24.06 | [M-H]^-^ | 283.0627 | 283.0612 | 5.3 | C16H12O5 | Calycosin | 283.0610;268.0379;239.0355;211.0410 | Astragali radix |
| 26 | 26.87 | [M+H]^+^ | 269.0804 | 269.0808 | -1.6 | C16H12O4 | Formononetin | 269.0797;253.0480;237.0538;213.0901;197.0585;  169.0635 | Astragali radix |
| 27 | 27.85 | [M+FA-H]^-^ | 825.4689 | 825.4642 | 5.7 | C42H68O13 | Saikosaponin A | 825.4684;779.4618;617.4083 | Bupleurum |
| 28 | 28.48 | [M+FA-H]^-^ | 825.4684 | 825.4642 | 5.1 | C42H68O13 | Saikosaponin B2 | 825.4689;779.4620;617.4039 | Bupleurum |
| 29 | 28.65 | [M+FA-H]^-^ | 825.4685 | 825.4642 | 5.2 | C42H68O13 | Saikosaponin D | 825.4717;779.4622;617.4057 | Bupleurum |
